# Lesion Length Impacts Long Term Outcomes of Drug-Eluting Stents and Bare Metal Stents Differently

**DOI:** 10.1371/journal.pone.0053207

**Published:** 2013-01-11

**Authors:** Shang-Hung Chang, Chun-Chi Chen, Ming-Jer Hsieh, Chao-Yung Wang, Cheng-Hung Lee, I-Chang Hsieh

**Affiliations:** Second Section of Cardiology and Percutaneous Coronary Intervention Center Department of Medicine, Chang Gung Memorial Hospital and Chang Gung University, Taipei, Taiwan; Sapienza University of Rome, Italy

## Abstract

**Background:**

Long lesions have been associated with adverse outcomes in percutaneous coronary interventions with bare metal stents (BMS). However, the exact impact of lesion length on the short- and long-term outcomes of drug-eluting stent (DES) implantations is not as clear.

**Methods and Results:**

This study compared the impact of lesion length on angiographic and clinical outcomes of BMS and DES in a single-center prospective registry. Lesion length was divided into tertiles. The primary endpoints were angiographically defined binary in-stent restenosis (ISR) rate and major adverse cardiac event (MACE). Of the 4,312 de novo lesions in 3,447 consecutive patients in the CAPTAIN registry, 2,791 lesions (of 2,246 patients) received BMS, and the remaining 1,521 lesions (of 1,201 patients) received DES. The mean follow-up duration was 4.5 years. The longer the lesion, the higher the ISR rate (14%, 18%, and 29%, p<0.001) and the lower the MACE-free survivals (p = 0.007) in the BMS group. However, lesion length showed no such correlation with ISR rates (4.7%, 3.3%, and 7.8%, p = 0.67) or MACE-free survivals (p = 0.19) in the DES group.

**Conclusions:**

In our single-center prospective registry, lesion length defined in tertiles has no impact on the short-term (ISR) or long-term (MACE) outcomes of patients implanted with DES. In contrast, longer lesion correlates with higher ISR and MACE rates in BMS group.

## Introduction

The management of long coronary lesions has become increasingly important in clinical practice because of the rising incidence of long or complex lesions in aging populations and their increasing comorbidity [1]. In-stent restenosis (ISR) is one of the main challenges in treating long lesions with stents while major adverse cardiac events (MACE) free survival is the gold standard for stents comparisons. Generally speaking, drug-eluting stents (DES) have been shown to be more efficacious than bare metal stents (BMS) in reducing ISR and MACE [2]–[5]. Stent length and lesion length have both been reported as very important predictors of ISR in the BMS era [6]–[9]. These two factors are thought to be less important in the DES era because DES reduce ISR dramatically in almost every type of lesion [10]–[11]. On the other hand, the effect of lesion length on the long term outcomes in the DES era has been ignored. A very recent study suggested that longer stents are associated with increased MACE rates at 1 year [12]. The exact difference in impact of lesion length on the long term outcomes for BMS and DES, however, is not clear. To bridge this gap, this study was conducted with the aim of comparing the real impact of lesion length on BMS and DES in terms of ISR and MACE-free survival. Data were collected from a prospectively created database, and angiographic follow-up was decided upon prior to data interpretation.

## Methods

### Subjects

The CAPTAIN (Cardiovascular Atherosclerosis and Percutaneous TrAnsluminal INterventions) registry is a physician-initiated prospective single-center observational study in a tertiary medical center, which enrolls consecutive patients undergoing stent implantation.

Both short and long term outcomes of stent implantations are examined in this paper. For short-term outcomes, a total of 4,745 consecutive patients with de novo native coronary artery lesions who had undergone successful emergency or elective percutaneous coronary intervention (PCI) at this hospital between November 1996 and December 2010 were registered. Patients were referred for coronary angiography based on angina, an abnormal stress test, or elevated markers of myocardial damage. Because of different timelines of restenosis between BMS and DES, follow-up angiographies were performed for 4,312 target lesions in 3,447 patients at either 6 months (in the BMS group) or 9 months (in the DES group) after the index procedure [13]–[18]. For long term outcomes, patients were scheduled to undergo clinical follow-up at 30 days, 6, 9, 12 months, and thereafter annually. The stents used in this study were either BMS (Palmaz-Schatz, Crown, Bx, Multi-link, Duet, Tristar, Penta, Pixel, Express, Liberte, S7, Driver, and Vision), or DES (Cypher, Taxus, Endeavor, Xience V). The BMS used in this study measured between 2.5 mm and 5 mm in diameter and between 7 mm and 38 mm in length. The DES used were between 2.25 and 4 mm in diameter and between 12 and 38 mm in length. After stent implantation, dual antiplatelet treatment of aspirin and a thienopyridine derivative (ticlopidine, 200 mg/day or clopidogrel, 75 mg/day) was to be maintained for 3–12 months. Thereafter, the decision regarding the duration of dual antiplatelet therapy was left to the discretion of each attending physician. Lifelong use of aspirin was suggested after the procedure except when a contraindication existed.

Lesions were classified into tertiles, and cut points were 14 and 21 mm for the BMS group and 16 and 24 mm for the DES group. If a patient had multiple stent implantations, the longest lesion was used in analysis.

### Definition of endpoints

The primary endpoints are binary ISR and MACE. Binary ISR at follow-up was defined as a stenosis occupying ≥50% of vessel diameter and occurring in the segment inside the stent or within a 5 mm segment proximal or distal to the stent. MACE was defined as a composite of cardiac death, ST-elevation and non–ST-elevation myocardial infarction (MI), coronary artery bypass grafting (CABG), or target lesion revascularization (TLR). An independent researcher unaware of the patient's treatment reviewed all clinical end points during follow-up. Lesion length was measured as the length of contiguous coronary narrowing (defined as percent diameter stenosis >50%) [19]. Angiographic variables derived from the index procedure and restudy, including absolute lesion length, stent length, reference vessel diameter, minimal luminal diameter, percent diameter stenosis, and late loss, were measured by automated edge detection or a digital caliber before and after stent deployment at baseline and follow-up coronary angiography, using the contrast-filled guiding catheter as a calibration reference [20]. A small vessel was defined as one having a pre-procedural reference diameter of less than 2.5 mm. Baseline clinical characteristics were collected during the index procedure. Lesions were qualitatively classified using the modified American College of Cardiology/American Heart Association grading system.

### Statistics

Categorical data were shown as percentages and compared between groups using chi-square. Continuous variables are presented as the mean ± standard deviation, and comparisons were made by analysis of variance (ANOVA). Spearman rank correlation was applied for association between ordinal variables. Cumulative curves for MACE were obtained using the Kaplan-Meier method and the groups were compared in terms of survival on log-rank tests. Data analysis was performed using STATA version 10 (StataCorp LP. College Station, TX, USA). A p value <0.05 was considered significant.

## Results

In a 14-year period, 3,447 patients were entered into a prospectively collated database. Angiographic follow-ups were 80% and 79% in the BMS and DES groups, respectively. ISR and late loss were assessed angiographically in 4,312 lesions (2,791 implanted with BMS and 1,521 with DES). MACE was followed in 3,447 patients (2,246 patients with BMS and 1,201 with DES). In both the BMS and DES groups, the patients had generally similar demographic and baseline clinical characteristics irrespective of lesion length, with the following exceptions: In the DES group, the middle subgroup had the lowest incidence of hypertension (Table 1). Lesion characteristics distribute similarly in the BMS and DES groups. The reference diameter of the target lesions in all subgroups for either BMS or DES was about 3.2 mm. In both BMS and DES groups, longer lesions were more calcified, more complex, and had been treated by multiple stents. The incidence of small vessels was generally very low in all subgroups (Table 2).

**Table 1:** Clinical characteristics.

	BMS (n = 2,246)				DES (n = 1,201)				BMS vs. DES (p)
Tertile (mm)	Shortest (<14) (n = 812)	Middle (14–21) (n = 825)	Longest (>21) (n = 609)	p	Shortest (<16) (n = 401)	Middle (16–24) (n = 411)	Longest (>24) (n = 389)	p	
Age (years)	61±10	61±11	60±11	NS	62±12	61±12	60±11	NS	60.8±0.2 vs. 60.9±0.3 (NS)
Male (%)	82	83	83	NS	81	84	81	NS	82.3 vs. 82 (NS)
Hypertension (%)	51	48	49	NS	60	52	59	0.04	50.7 vs. 57 (<0.001)
Diabetes (%)	23	22	22	NS	26	26	28	NS	23 vs. 27.3 (0.003)
Smoking (%)	50	49	50	NS	38	42	41	NS	49.6 vs. 40.7 (<0.001)
Dyslipidemia (%)	58	60	62	NS	53	55	54	NS	59.5 vs. 53.8 (0.001)
% of diseased coronary arteries				0.02				0.02	(NS)
1-vessel	37	43	43		40	46	39		40.7 vs. 41.6
2-vessel	34	33	32		33	31	35		33.1 vs. 33
3-vessel	26	23	24		24	22	25		24.5 vs. 23.77
Left main	3	1	1		3	1	1		1.6 vs. 1.6

Values are mean ± SD where appropriate. BMS, bare metal stent; DES, drug-eluting stent NS, not significant.

**Table 2:** Lesions and Procedural Characteristics.

	BMS (n = 2,791)				DES (n = 1,521)				BMS vs. DES (p)
Tertile (mm)	Shortest (<14) (n = 906)	Middle (14–21) (n = 1,012)	Longest (>21) (n = 873)	P	Shortest (<16) (n = 447)	Middle (16–24) (n = 519)	Longest (>24) (n = 555)	P	
Lesion length (mm)	12.1±2.2	16.9±2.2	29.4±8.8	<0.0001	12.6±2.2	19.1±1.9	33.8±13	<0.0001	18.3±0.2 vs. 22.6±0.3 (<0.001)
Reference (mm)	3.2±0.5	3.2±0.5	3.1±0.5	NS	3.2±.05	3.2±0.4	3.2±0.4	NS	3.18±0.01 vs. 3.19±0.01 (NS)
Multiple stents (%)	0	2	23	<0.0001	0	1	34	<0.0001	8 vs. 12.9 (<0.001)
AHA type B2+C (%)	60	74	97	<0.0001	71	82	98	<0.0001	76.9 vs. 84.6 (<0.001)
Lesion site (%)				<0.0001				<0.0001	NS
Left Main	4	1	1		4	1	2		2.2 vs. 2.2
LAD	44	45	51		44	50	51		47.7 vs. 50.8
LCX	18	19	12		22	19	12		17.1 vs. 17.8
RCA	29	33	34		23	26	34		33 vs. 29.3
Calcification (%)	13	13	25	<0.0001	12	11	16	0.04	16.7 vs. 12.9 (0.001)
Angulated >45° (%)	7	6	7	NS	3	2	2	NS	6.7 vs. 2.1 (<0.001)
Eccentric (%)	58	49	36	<0.0001	46	43	31	<0.0001	48 vs. 39.6 (<0.001)
Small vessel (%)	8	4	5	0.005	3	4	1	0.003	5.8 vs. 2.7 (0.001)
Bifurcation lesion (%)	11	10	10	NS	11	11	10	NS	10 vs. 10.1 (NS)
Ostial lesion (%)	9	4	6	0.001	14	7	8	<0.0001	6.1 vs. 9.6 (<0.001)
Chronic total occlusion (%)	5	12	22	<0.0001	10	24	24	<0.0001	13 vs. 20 (<0.001)
Angiographic follow up									
Late loss (mm)	0.9±0.6	1±0.66	1.2±0.7	<0.0001	0.38±0.58	0.35±0.56	0.51±0.7	<0.0001	1.05±0.01 vs. 0.42±0.02 (<0.001)
Restenosis (%)	14	18	29	<0.0001	4.7	3.3	7.8	0.004	20.3 vs. 5.3 (<0.001)

BMS, bare metal stent; DES, drug-eluting stent; LAD, left anterior descending; LCX, left circumflex; RCA, right coronary artery.

**Figure 1:**
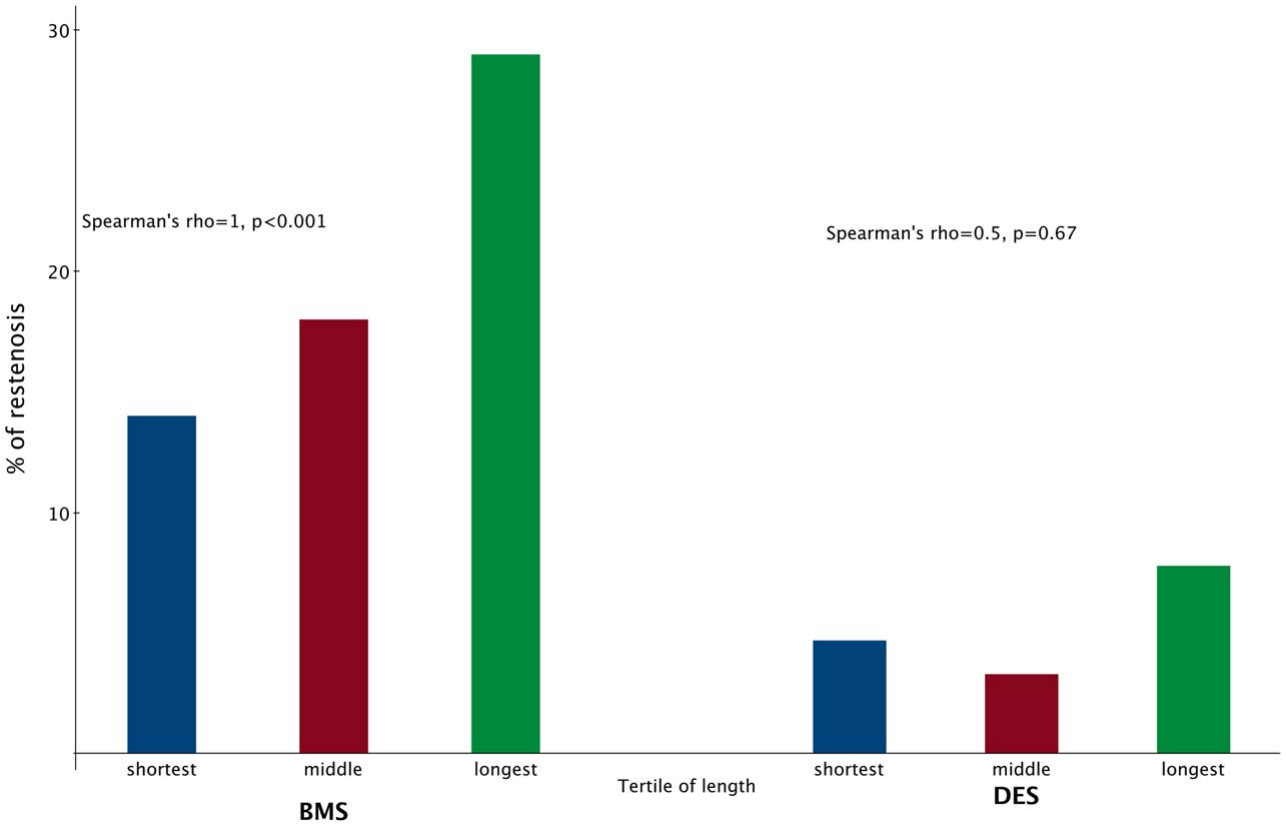
Intra-stent restenosis rate defined by scheduled angiographic follow-up of various lesion length. BMS: bare metal stent. DES: drug-eluting stents.

The overall angiographic ISR rate was much higher for the BMS group than for the DES group (20.3% vs. 5.3%, p<0.001). In the BMS group, the ISR rate correlated perfectly with lesion length (14%, 18%, and 29%, Spearman's rho = 1, p<0.001) (Figure 1). However, ISR rates showed no such correlation with lesion length in the DES group (4.7%, 3.3%, and 7.8%, Spearman's rho = 0.5, p = 0.67).

**Figure 2:**
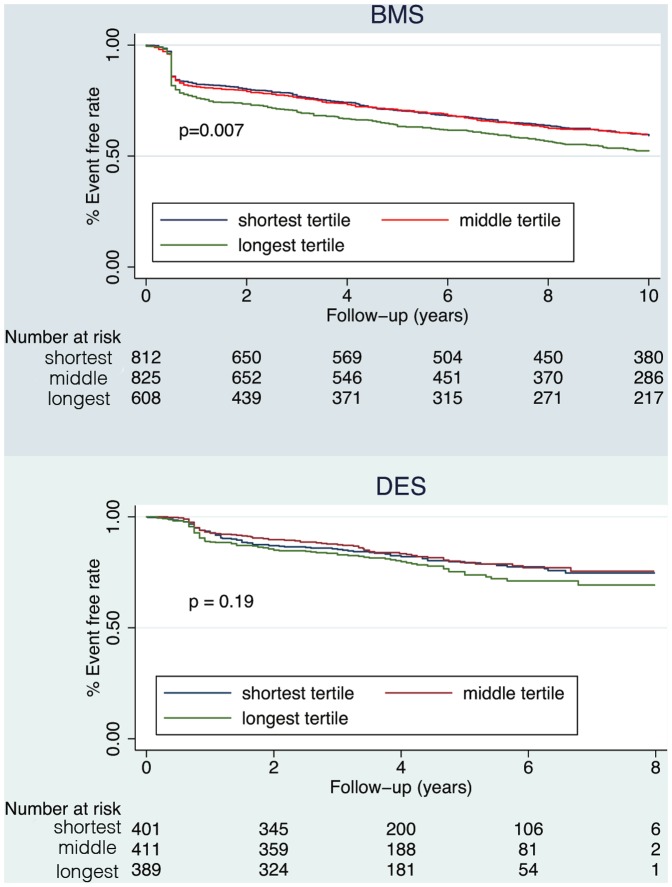
Kaplan-Meier estimates: major adverse cardiac events (MACE) free survival of patients with various lesion length. BMS: bare metal stent. DES: drug-eluting stents.

On chronic results, BMS and DES patients were both divided into tertiles based on lesion length. The BMS patients were followed up for ten years, and the DES patients were followed up for eight years. The survival curve for the BMS group shows that lesion length affected survival rates (p = 0.007). On the other hand, the survival rates of DES patients did not differ among the lesion length tertiles (p = 0.19) (Figure 2).

## Discussion

This study presents three major findings. First, the DES group had much lower ISR and better MACE-free survival than the BMS group at any lesion length. Second, ISR rates correlated perfectly and positively with lesion lengths in the BMS group. However, lesion length had no such correlation to ISR in the DES group. Finally, longest lesions had the worst long-term MACE-free survival in the BMS group while such results were not as pronounced in the DES group.

Lesion length and stent length have been reported as important predictors of ISR for various types of BMS and DES [19]. From a very early stage of coronary intervention, studies have shown that shorter BMSs were associated with fewer clinical events and lower ISR rates [21]. Stented segment length was also found to be an important and independent predictor of restenosis when using various types of BMS in more than 1000 lesions [6]. Kereiakes et al, in their meta-analysis of 4 multi-link stent trials, described a fairly linear correlation between stent lengths and IRS rates for stents in 6 length groups [22]. That assertion is supported by the present study, in which we also observed that in the BMS group, the longer the lesion length, the higher the ISR rates. Similarly, stent length and lesion length have been reported as independent predictors of IRS in various DES such as sirolimus-eluting stents [23]–[25]. However, in the present study we did not see the same result. According to our data, lesion length has no significant effect on ISR rate for DES until lesions are longer than 24 mm.

Several studies have suggested that longer lesions were associated with higher MACE rates in the BMS and DES eras [12], [26], [27]. Our results showed similar results in the BMS group, but not in the DES group. Although different populations, follow-up protocols, and definitions of endpoints prevented direct comparisons between this and these observational studies, our study is distinctive in its very long follow-up time frame. In both BMS and DES groups, most of the MACEs were contributed mainly by TLR performed in scheduled angiography. The impact of lesion length on MACE in BMS would have been much smaller if only “hard” endpoints such as cardiac deaths and myocardial infractions had been considered as endpoints.

To our knowledge, this report is the first one that has directly compared IRS or very long term MACE-free survival between BMS and DES for every lesion length subgroups of a single registry. DES decreases IRS more dramatically than does BMS [23], [28]. For example, Dawkins reported 12% vs. 36% ISR rates of DES vs. BMS in a TAXUS VI trial [29]. The present study supports these findings. Most data from numerous trials regarding lesion length and ISR in the DES era showed that DES resolved the issue of restenosis in lesions of various lengths [30], [31]. Again, this paper supports that conclusion, but only for lesions that are shorter than 24 mm. DES is still superior to BMS in lesions longer than 24 mm, but the advantage is not as great as in shorter lesions.

The results of this study may have some clinical implications for the daily practice of interventional cardiologists. DES has lessened the impact of lesion length on ISR rate and MACE-free survival to some degree; therefore operators might feel comfortable in deploying DES for long lesions. One should be aware that some long lesions in this study (23% in BMS and 34% in DES) were covered by multiple overlapping stents, implying that DES were more effective in treating long lesions even when multiple stenting was involved. For example, Räber et al. reported a relatively high (18%) target lesion revascularization rate in 333 patients who had received multiple and overlapping DES [32]. However, before further evidence becomes available, one may argue that lesion length per se—instead of being a true underlying reason for higher ISR rate in the DES group—might merely be a surrogate for many other factors such as severity of disease, flow reserve, local inflammation, or lesion complexity. For example, the extent of intimal hyperplasia is significantly greater in lesions treated with longer stents [33]. Therefore, more studies regarding the pathological effects of lesion length are needed. Nevertheless, lesion length is still a convenient parameter for making clinical decisions and predicting outcomes.

The present study has several limitations that should be mentioned. First, this is a registry observation from a single center, so the choice of stents and follow-up angiography might be biased considerably by operators, patients, or the availability of devices. Second, optimal medication choices, especially antiplatelet therapy, the technique and concept of percutaneous coronary intervention, and the design of devices, were evolving greatly during the long period of this study, the effect of which this analysis did not take into account. The difference of ticlopidine and clopidogrel, however, were examined, and it showed no effects on MACE. (Figure S1 of supplement data) Third, a major consideration that should be emphasized is the heterogeneity of DES. Recent randomized trials have shown that second-generation DES are superior to first-generation DES (especially palitaxel-eluting stents) in their ability to lower incidence of restenosis [34]. However, the very long term follow-up frame might have partially compensated for these limitations. The ‘all comers’ design of this study, which included a variety of stents, lesions, and patients, should be able to reflect the true impact of lesion length in the real world.

In conclusion, lesion length has different effects on ISR rates and MACE-free survival in BMS and DES in the real world. Lesion length positively associates with ISR rate for BMS,and longest lesions have the worst MACE-free survival. DES considerably lowers the effects of lesion length on ISR rates and MACE-free survival.

## Supporting Information

Figure S1 MACE free survival of BMS group before and after clopidogrel era were similar. (Logrank p = 0.5). Blue line indicates the survival curve of patients treated with ticlopidine while red line indicates the curve of patients treated with clopidogrel.
(DOC)

## References

[1] Boden WE, O'rourke RA, Teo KK, Hartigan PM, Maron DJ, et al (2007) The evolving pattern of symptomatic coronary artery disease in the United States and Canada: baseline characteristics of the Clinical Outcomes Utilizing Revascularization and Aggressive DruG Evaluation (COURAGE) trial. Am J Cardiol 99: 208–212. 17223420 10.1016/j.amjcard.2006.07.082

[2] Liistro F, Stankovic G, Di Mario C, Takagi T, Chieffo A, et al (2002) First clinical experience with a paclitaxel derivate-eluting polymer stent system implantation for in-stent restenosis: immediate and long-term clinical and angiographic outcome. Circulation 105: 1883–1886. 11997271 10.1161/01.cir.0000016042.69606.61

[3] Koh AS, Chia S, Choi LM, Sim LL, Chua TSJ, et al (2011) Long-term outcomes after coronary bare-metal-stent and drug-eluting-stent implantations: a ‘real-world’ comparison among patients with diabetes with diffuse small vessel coronary artery disease. Coronary Artery Disease 22: 96–99. 21164343 10.1097/MCA.0b013e32834236d0

[4] Violini R, Musto C, De Felice F, Nazzaro MS, Cifarelli A, et al (2010) Maintenance of long-term clinical benefit with sirolimus-eluting stents in patients with ST-segment elevation myocardial infarction 3-year results of the SESAMI (sirolimus-eluting stent versus bare-metal stent in acute myocardial infarction) trial. J Am Coll Cardiol 55: 810–814. 20170821 10.1016/j.jacc.2009.09.046

[5] Menichelli M, Parma A, Pucci E, Fiorilli R, De Felice F, et al (2007) Randomized trial of Sirolimus-Eluting Stent Versus Bare-Metal Stent in Acute Myocardial Infarction (SESAMI). J Am Coll Cardiol 49: 1924–1930. 17498576 10.1016/j.jacc.2007.01.081

[6] Kobayashi Y, De Gregorio J, Kobayashi N, Akiyama T, Reimers B, et al (1999) Stented segment length as an independent predictor of restenosis. J Am Coll Cardiol 34: 651–659. 10483943 10.1016/s0735-1097(99)00303-4

[7] Kastrati A, Elezi S, Dirschinger J, Hadamitzky M, Neumann FJ, et al (1999) Influence of lesion length on restenosis after coronary stent placement. Am J Cardiol 83: 1617–1622. 10392864 10.1016/s0002-9149(99)00165-4

[8] Mehran R, Dangas G, Abizaid AS, Mintz GS, Lansky AJ, et al (1999) Angiographic patterns of in-stent restenosis: classification and implications for long-term outcome. Circulation 100: 1872–1878. 10545431 10.1161/01.cir.100.18.1872

[9] de Feyter PJ, Kay P, Disco C, Serruys PW (1999) Reference chart derived from post-stent-implantation intravascular ultrasound predictors of 6-month expected restenosis on quantitative coronary angiography. Circ 100: 1777–1783. 10.1161/01.cir.100.17.1777 10534464

[10] Rozenman Y, Witzling V, Tamari I, Turkisher V, Kriviski M, et al (2009) Impact of stent length on restenosis in patients with acute myocardial infarction treated with primary percutaneous coronary intervention: analysis based on data from the Trial to Assess the Use of the Cypher Stent in Acute Myocardial Infarction Treated with Balloon Angioplasty (TYPHOON). EuroIntervention 5: 219–223. 19527979 10.4244/eijv5i2a34

[11] Rathore S, Terashima M, Katoh O, Matsuo H, Tanaka N, et al (2009) Predictors of angiographic restenosis after drug eluting stents in the coronary arteries: contemporary practice in real world patients. EuroIntervention 5: 349–354. 19736160 10.4244/v5i3a55

[12] Caputo RP, Goel A, Pencina M, Cohen DJ, Kleiman NS, et al (2012) Impact of Drug Eluting Stent Length on Outcomes of Percutaneous Coronary Intervention (from the EVENT Registry). The American journal of cardiology 110: 350–355. 22560770 10.1016/j.amjcard.2012.03.031

[13] Cutlip DE, Chauhan MS, Baim DS, Ho KKL, Popma JJ, et al (2002) Clinical restenosis after coronary stenting: perspectives from multicenter clinical trials. J Am Coll Cardiol 40: 2082–2089. 12505217 10.1016/s0735-1097(02)02597-4

[14] Kimura T, Abe K, Shizuta S, Odashiro K, Yoshida Y, et al (2002) Long-term clinical and angiographic follow-up after coronary stent placement in native coronary arteries. Circ 105: 2986–2991. 10.1161/01.cir.0000019743.11941.3b 12081992

[15] Hsieh I-C, Huang H-L, See L-C, Chang S-H, Chang H-J, et al (2006) Improvement in left ventricular function following coronary stenting in patients with acute myocardial infarction: 6-month and 3-year follow-up. International journal of cardiology 111: 209–216. 16188332 10.1016/j.ijcard.2005.07.005

[16] Kimura T, Yokoi H, Nakagawa Y, Tamura T, Kaburagi S, et al (1996) Three-year follow-up after implantation of metallic coronary-artery stents. N Engl J Med 334: 561–566. 8569823 10.1056/NEJM199602293340903

[17] Teirstein PS (2010) Drug-eluting stent restenosis: an uncommon yet pervasive problem. Circ 122: 5–7. 10.1161/CIRCULATIONAHA.110.962423 20566948

[18] Park KW, Kim C-H, Lee H-Y, Kang H-J, Koo B-K, et al (2010) Does “late catch-up” exist in drug-eluting stents: insights from a serial quantitative coronary angiography analysis of sirolimus versus paclitaxel-eluting stents. Am Heart J 159: 446–453.e443. 20211308 10.1016/j.ahj.2010.01.001

[19] Mauri L, O'Malley AJ, Cutlip DE, Ho KKL, Popma JJ, et al (2004) Effects of stent length and lesion length on coronary restenosis. Am J Cardiol 93: 1340–1346, A1345. 15165911 10.1016/j.amjcard.2004.02.027

[20] Hsieh I-C, Chang H-J, Huang H-L, See L-C, Chern M-S, et al (2004) Acute and long-term clinical and angiographic outcomes of coronary stenting using Palmaz-Schatz stent and ACS Multi-Link stent. Catheter Cardiovasc Interv 62: 453–460. 15274153 10.1002/ccd.20116

[21] Foley DP, Pieper M, Wijns W, Suryapranata H, Grollier G, et al (2001) The influence of stent length on clinical and angiographic outcome in patients undergoing elective stenting for native coronary artery lesions; final results of the Magic 5L Study. Eur Heart J 22: 1585–1593. 11492988 10.1053/euhj.2001.2752

[22] Kereiakes D, Linnemeier TJ, Baim DS, Kuntz R, O'Shaughnessy C, et al (2000) Usefulness of stent length in predicting in-stent restenosis (the MULTI-LINK stent trials). Am J Cardiol 86: 336–341. 10922447 10.1016/s0002-9149(00)00928-0

[23] Moses JW, Leon MB, Popma JJ, Fitzgerald PJ, Holmes DR, et al (2003) Sirolimus-eluting stents versus standard stents in patients with stenosis in a native coronary artery. N Engl J Med 349: 1315–1323. 14523139 10.1056/NEJMoa035071

[24] Holmes DR, Leon MB, Moses JW, Popma JJ, Cutlip D, et al (2004) Analysis of 1-year clinical outcomes in the SIRIUS trial: a randomized trial of a sirolimus-eluting stent versus a standard stent in patients at high risk for coronary restenosis. Circulation 109: 634–640. 14769686 10.1161/01.CIR.0000112572.57794.22

[25] Mauri L, O'Malley AJ, Popma JJ, Moses JW, Leon MB, et al (2005) Comparison of thrombosis and restenosis risk from stent length of sirolimus-eluting stents versus bare metal stents. Am J Cardiol 95: 1140–1145. 15877983 10.1016/j.amjcard.2005.01.039

[26] Tcheng JE, Lim IH, Srinivasan S, Jozic J, Gibson CM, et al (2009) Stent parameters predict major adverse clinical events and the response to platelet glycoprotein IIb/IIIa blockade: findings of the ESPRIT trial. Circ Cardiovasc Interv 2: 43–51. 20031692 10.1161/CIRCINTERVENTIONS.108.809285

[27] Shirai S, Kimura T, Nobuyoshi M, Morimoto T, Ando K, et al (2010) Impact of multiple and long sirolimus-eluting stent implantation on 3-year clinical outcomes in the j-Cypher Registry. JACC Cardiovasc Interv 3: 180–188. 20170875 10.1016/j.jcin.2009.11.009

[28] Dibra A, Kastrati A, Alfonso F, Seyfarth M, Pérez-Vizcayno M-J, et al (2007) Effectiveness of drug-eluting stents in patients with bare-metal in-stent restenosis: meta-analysis of randomized trials. J Am Coll Cardiol 49: 616–623. 17276188 10.1016/j.jacc.2006.10.049

[29] Dawkins KD, Grube E, Guagliumi G, Banning AP, Zmudka K, et al (2005) Clinical efficacy of polymer-based paclitaxel-eluting stents in the treatment of complex, long coronary artery lesions from a multicenter, randomized trial: support for the use of drug-eluting stents in contemporary clinical practice. Circulation 112: 3306–3313. 16286586 10.1161/CIRCULATIONAHA.105.552190

[30] Kim Y-H, Park S-W, Lee S-W, Park D-W, Yun S-C, et al (2006) Sirolimus-eluting stent versus paclitaxel-eluting stent for patients with long coronary artery disease. Circulation 114: 2148–2153. 17060388 10.1161/CIRCULATIONAHA.106.666396

[31] Grube E, Dawkins K, Guagliumi G, Banning A, Zmudka K, et al (2009) TAXUS VI final 5-year results: a multicentre, randomised trial comparing polymer-based moderate-release paclitaxel-eluting stent with a bare metal stent for treatment of long, complex coronary artery lesions. EuroIntervention 4: 572–577. 19378676 10.4244/eijv4i5a97

[32] Räber L, Jüni P, Löffel L, Wandel S, Cook S, et al (2010) Impact of stent overlap on angiographic and long-term clinical outcome in patients undergoing drug-eluting stent implantation. J Am Coll Cardiol 55: 1178–1188. 20298923 10.1016/j.jacc.2009.11.052

[33] Kang S-J, Mintz GS, Park D-W, Lee S-W, Kim Y-H, et al (2011) Mechanisms of in-stent restenosis after drug-eluting stent implantation: intravascular ultrasound analysis. Circ Cardiovasc Interv 4: 9–14. 21266707 10.1161/CIRCINTERVENTIONS.110.940320

[34] Dangas GD, Claessen BE, Caixeta A, Sanidas EA, Mintz GS, et al (2010) In-stent restenosis in the drug-eluting stent era. J Am Coll Cardiol 56: 1897–1907. 21109112 10.1016/j.jacc.2010.07.028

