# Supplementary material for: Lesion Length Impacts Long Term Outcomes of Drug-Eluting Stents and Bare Metal Stents Differently
Source: PLoS One. 2013 Jan 11;8(1):e53207. doi: 10.1371/journal.pone.0053207 (PMC3543456; doi:10.1371/journal.pone.0053207)

Figure 1

MACE free survival of BMS group before and after clopidogrel era were similar. (Logrank p = 0.5). Blue line indicates the survival curve of patients treated with ticlopidine while red line indicates the curve of patients treated with clopidogrel.


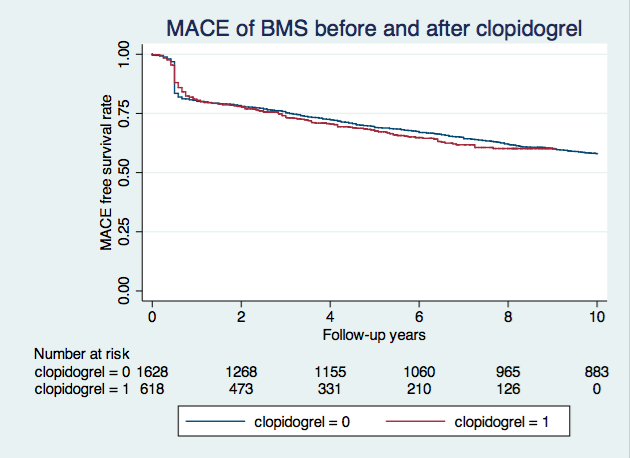

Supplement: Figure S1 — MACE free survival of BMS group before and after clopidogrel era were similar. (Logrank p = 0.5). Blue line indicates the survival curve of patients treated with ticlopidine while red line indicates the curve of patients treated with clopidogrel. (DOC) [file pone.0053207.s001.doc]
